# Prevalence of vitamin D deficiency and associated risk of all-cause and cause-specific mortality among middle-aged and older adults in the United States

**DOI:** 10.3389/fnut.2023.1163737

**Published:** 2023-05-18

**Authors:** Ting-Yi Wang, Hung-Wei Wang, Ming-Yan Jiang

**Affiliations:** ^1^Renal Division, Department of Internal Medicine, Sin-Lau Hospital, Tainan, Taiwan; ^2^Renal Division, Department of Internal Medicine, Chi Mei Hospital Chiali, Tainan, Taiwan; ^3^Renal Division, Department of Internal Medicine, Chi Mei Medical Center, Tainan, Taiwan; ^4^Department of Pharmacy, Chia Nan University of Pharmacy and Science, Tainan, Taiwan

**Keywords:** vitamin D deficiency, vitamin D insufficiency, prevalence, mortality, pneumonia

## Abstract

**Introduction:**

The prevalence of vitamin D deficiency varied among populations and regions worldwide. In addition, the association between vitamin D deficiency and health outcomes remained controversial. Our study aimed to investigate the prevalence of vitamin D deficiency and its association with mortality risk among non-institutional middle-aged and older adults in the United States.

**Method:**

The study population included 11,119 adult participants aged between 50 and 79 years in the 2007–2016 National Health and Nutrition Examination Survey (NHANES). Vitamin D status was divided as ≤ 30 (severely deficient), 30.1–50 (moderately deficient), 50.1–75 (insufficient), 75.1–100 (sufficient), and > 100 nmol/L (very sufficient). NHANES data were linked to National Death Index to ascertain the survival status and cause of death.

**Results:**

The population aged 61.5 years (survey-weighted) and 47.9% were men. Among them, 4.6% were severely vitamin D deficient, 15.2% moderately deficient, and 33.6% insufficient. Individuals with higher vitamin D levels tended to be female, older, white people, non-smoker, non-single, more educated, with higher family income, and lower body mass index. During a median follow-up of 97.0 months, a total of 1,585 participants died (15.9 per 10,000 person-months). The crude analysis showed that vitamin D deficiency, but not vitamin D insufficiency, correlated to higher all-cause mortality risk. The association remained similar after adjusting for potential confounders, showing that vitamin D deficiency (HR: 1.38, 95% CI 1.15–1.66), but not vitamin D insufficiency (HR: 1.03, 95% CI 0.88–1.20), correlated to higher all-cause mortality risk. In addition, we showed that vitamin D deficiency was an independent risk factor for death from pneumonia (HR: 3.82, 95% CI 1.14–12.86) but not from cardiovascular diseases, cancer, or cerebrovascular diseases.

**Conclusion:**

In summary, among middle-aged and older adults in the United States, nearly 20% were vitamin D deficient. Vitamin D deficiency, but not vitamin D insufficiency, correlated to increased mortality risk.

## Introduction

Vitamin D exerts pleiotropic effects in the human body involved in calcium homeostasis, bone metabolism, and regulation of cardiovascular function (1). Several epidemiological studies have shown that low serum vitamin D status was associated with increased risks of various chronic illnesses such as cardiovascular diseases, diabetes, cancer, and neuropsychiatric disorders (2). In addition, vitamin D is an immunomodulatory hormone that regulates multiple components of the innate or adaptive immune system (3), and vitamin D deficiency has been recognized as a risk factor for respiratory tract infection (4–6). Among hospitalized patients with community-acquired pneumonia, evidence also suggested that those with vitamin D deficiency were more likely to have more severe disease and a greater risk of mortality when compared to those with higher vitamin D levels (7, 8).

Vitamin D deficiency is a common health problem worldwide. Although a low vitamin D status is uncommon in most developed countries, literature studies have demonstrated that subclinical vitamin D deficiency can exist in certain populations such as older adults (9–11). In addition, while studies suggest that vitamin D deficiency may play roles in downstream adverse health consequences, the beneficial effects of vitamin D supplementation on health outcomes remain inconclusive, and the causal relationship between vitamin D and health outcomes is still under debate (12–14). Accordingly, our study aims to assess the prevalence of vitamin D deficiency and the secular trend among non-institutional middle-aged and older adults in the United States and to investigate the association of vitamin D deficiency with mortality risk.

## Materials and methods

### Data source

We obtained data from the National Health and Nutrition Examination Survey (NHANES) of the United States, which is a series of health-related programs conducted by the National Center for Health Statistics. NHANES constitutes a series of cross-sectional, multistage probability sampling for civilian noninstitutionalized population across the United States.^1^ NHANES data were collected from survey participants using questionnaires on health-related topics at participants’ homes and a physical examination and laboratory tests in a mobile examination center, with data released in 2-year cycles. The data were available for public use on the website of the National Center for Health Statistics (Available at: https://wwwn.cdc.gov/nchs/nhanes/Default.aspx). All NHANES protocols were approved by the research ethics review board of the National Center for Health Statistics, and all the participants provided written informed consent.

### Study population

**Figure 1:**
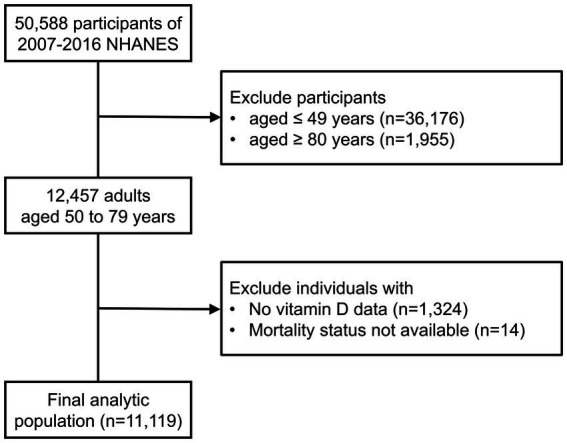
Population selection flowchart.

In this retrospective cohort study, we merged the data from 5 discrete 2-year cycles (2007–2008 through 2015–2016) of the continuous NHANES (*N* = 50,588). We restricted our study population to 2007 through 2016 NHANES cycles to maintain the consistency for vitamin D measurement. We included individuals aged from 50 to 79 years at the time of examination (*n* = 12,457). Because individuals aged 80 and over are topcoded at 80 years of age, we excluded these participants from our analysis. After excluding individuals without data on vitamin D levels (*n* = 1,324) or survival status (*n* = 14), a total of 11,119 participants were included in the analysis (Figure 1).

### Exposure

Serum levels of vitamin D, including 25-hydroxyvitamin D2 and 25-hydroxyvitamin D3, were measured by high-performance liquid chromatography–tandem mass spectrometry (HPLC-MS/MS). Because of some controversial definitions of vitamin D deficiency, we defined vitamin D levels of ≤ 30 nmol/L as severely deficient, 30.1–50 nmol/L as moderately deficient, 50.1–75 nmol/L as insufficient, 75.1–100 nmol/L as sufficient, and > 100 nmol/L as very sufficient according to previous studies (15, 16).

### Outcome

The NHANES data were linked to death records from the National Death Index (NDI) to ascertain survival status through probabilistic matching and death certificate review. International Classification of Diseases–Tenth Revision was used to define the cause of death. Deaths due to numerous causes were identified according to the leading causes of death included in the publicly available NHANES linked mortality file. Death from cardiovascular diseases (CVD) was defined by leading causes of death coded as I00–I09, I11, I13, I20–I51, from cancer as C00–C97, from cerebrovascular diseases (CVA) as I60–I69, and from pneumonia as J09–J18. The follow-up period for each participant is the time between the date of the NHANES baseline interview and the date of the participant’s death or the last date of follow-up (December 31, 2019), whichever came first.

### Covariates

Self-identified race/ethnicity was categorized as non-Hispanic white people, non-Hispanic black people, Hispanics, and other races including multi-racial. Educational level was dichotomized into those with high school education and some college or above. Marital status was dichotomized into married or living with a partner (non-single), and widowed, divorced, separated, or never married (single). The ratio of family income to poverty (PIR) was calculated by dividing total family income by the poverty threshold specific to family size and the appropriate year and state. We classified PIR into three categories: < 1.3 (low income), ≥ 1.3–< 3.5 (middle income), and ≥ 3.5 (high income). Survey month was dichotomized into November 1–April 30 and May 1–October 31 as reported by NHANES. Body mass index (BMI) was calculated as body weight in kilograms divided by the square of height in meters and was classified into three categories: < 25 (normal), ≥ 25 to < 30 (overweight), and ≥ 30 kg/m^2^ (obese). Diabetes and hypertension were defined by self-reporting diagnosis of the disease or taking medications. Cardiovascular disease (CVD) was defined by self-reporting a history of congestive heart failure, coronary heart disease, angina, or heart attack; previous stroke was also defined by self-reporting a history of the diseases.

### Statistical analysis

The characteristics of the sample population were described using survey-weighted means and standard errors (SE) or counts and survey-weighted proportions. We used survey-weighted linear regression analysis to test the secular trend in the prevalence of various vitamin D statuses. We also performed a survey-weighted linear regression analysis to explore the association of covariates with serum vitamin D levels. We combined serum vitamin D levels of ≤ 30 nmol/L and 30.1–50 nmol/L as vitamin D deficiency and levels of 75.1–100 nmol/L and > 100 nmol/L as vitamin D sufficiency for survival analysis, using the Kaplan–Meier method with Log-Rank test. Survey-weighted Cox regression analysis was performed to explore the association between vitamin D status and mortality risk with adjustment for potential confounders. In model 1, we adjusted for age, sex, and survey month. In model 2, we added self-identified race/ethnicity, BMI, diabetes, hypertension, CVD, previous stroke, and smoking status in addition to the variables in model 1. In model 3, we further added socioeconomic factors including marital status, educational level, and the ratio of family income to poverty into the regression model. Data were presented as hazard ratio (HR) and 95% confidence interval (CI). The proportional hazards assumption was tested by including time-dependent covariates in the Cox model and showed no violation of the assumption. Because the distribution of vitamin D levels might not be normal, we also performed survey-weighted multinomial logistic regression analysis to explore the predictors of vitamin D deficiency or insufficiency using the vitamin D sufficient group as the reference. Data were presented as odds ratio (OR) and 95% confidence interval (CI). Statistical computation was performed using SAS 9.4.

## Results

**Figure 2:**
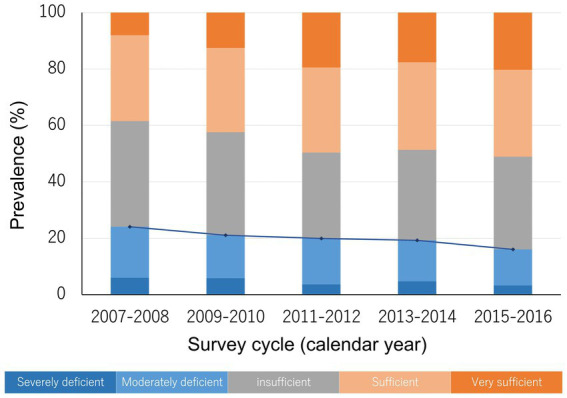
The prevalence of different vitamin D status and the secular trend. The blue line indicates the trend in the prevalence of vitamin D deficiency (severe deficiency plus moderate deficiency).

The weighted age (mean ± standard error) of the population was 61.5 ± 0.1 years old and 47.9% of them were men, with race/ethnicity distribution of 74.6% white people, 9.7% black people, 9.4% Hispanics, and 6.3% other races/ethnicities. Overall, the prevalence of severe and moderate vitamin D deficiency was 4.6 and 15.2%, respectively, while less than half of the population was sufficient in serum vitamin D levels (Table 1). When we stratified the population by sex, the prevalence of vitamin D deficiency did not differ markedly between men and women. In addition, when stratified by age groups, we found that more than 20% of people in their 50s were vitamin D deficient, with lower prevalence among people in their 60s and 70s. Additionally, while the prevalence of vitamin D deficiency in non-Hispanic white people was close to 10%, it was much higher in Hispanics and non-Hispanic black people, at nearly 30 and 50%, respectively. Furthermore, we observed that the prevalence of vitamin D deficiency was higher among those who were less educated, single, had lower family income, obese, current smokers, and those with a history of diabetes, hypertension, cardiovascular disease, or stroke. When stratified by survey cycles, we observed that the secular trend in the prevalence of vitamin D deficiency was declining (Table 1; Figure 2).

**Table 1:** Survey-weighted characteristics of the sample population by vitamin D status.

	Severely deficient	Moderately deficient	Insufficient	Sufficient	Very sufficient	*p*-value
Vit D level (nmol/L)	≤ 30	30.1–50	50.1–75	75.1–100	>100	
Number of participants	788 (4.6%)	2,333 (15.2%)	3,978 (33.6%)	2,786 (30.5%)	1,234 (16.0%)	
Sex						< 0.001
Male	359 (4.0%)	1,202 (15.2%)	2,183 (39.1%)	1,336 (31.5%)	432 (10.2%)	
Female	429 (5.1%)	1,131 (15.3%)	1795 (28.7%)	1,450 (29.6%)	802 (21.4%)	
Age (years old)	60.2 ± 0.4	60.4 ± 0.2	60.8 ± 0.2	62.1 ± 0.2	63.2 ± 0.3	< 0.001
Age group						< 0.001
50–59	361 (5.6%)	968 (17.0%)	1,564 (36.7%)	928 (27.9)	329 (12.8%)	
60–69	288 (3.7%)	937 (14.7%)	1,520 (31.7%)	1,054 (32.9%)	481 (17.1%)	
70–79	139 (3.8%)	428 (12.0%)	894 (29.8%)	804 (32.6%)	424 (21.8%)	
Race						< 0.001
White people	152 (2.7%)	603 (11.5%)	1,594 (32.7%)	1,576 (34.4%)	779 (18.8%)	
Black people	397 (16.8%)	747 (30.6%)	730 (29.3%)	393 (16.2%)	168 (7.1%)	
Hispanics	184 (6.5%)	762 (25.4%)	1,282 (43.7%)	556 (19.2%)	160 (5.2%)	
Others	55 (5.7%)	221 (20.4%)	372 (36.7%)	261 (24.0%)	127 (13.2%)	
Educational attainment						< 0.001
≤ high school	456 (6.0%)	1,395 (18.9%)	2,159 (34.6%)	1,306 (28.1%)	503 (12.5%)	
≥ some college	332 (3.7%)	936 (12.8%)	1817 (33.0%)	1,478 (32.1%)	729 (18.4%)	
Marital status^#^						< 0.001
Non-single	386 (3.4%)	1,333 (13.3%)	2,550 (34.4%)	1850 (33.0%)	748 (15.9%)	
Single	401 (7.1%)	999 (19.3%)	1,427 (32.1%)	933 (25.2%)	486 (16.4%)	
PIR	2.33 ± 0.08	2.75 ± 0.08	3.19 ± 0.06	3.51 ± 0.06	3.64 ± 0.07	< 0.001
PIR group						< 0.001
< 1.3	275 (7.9%)	807 (23.3%)	1,151 (36.0%)	654 (23.0%)	240 (9.8%)	
1.3 to < 3.5	281 (5.9%)	786 (16.3%)	1,340 (34.8%)	875 (28.2%)	407 (14.9%)	
≥ 3.5	145 (2.2%)	516 (11.5%)	1,121 (32.4%)	1,003 (34.7%)	490 (19.1%)	
BMI (kg/m^2^)	31.6 ± 0.4	31.4 ± 0.3	30.0 ± 0.2	29.0 ± 0.2	27.6 ± 0.2	< 0.001
BMI group						< 0.001
BMI < 25	154 (4.0%)	445 (11.1%)	797 (26.7%)	757 (34.2%)	415 (23.9%)	
BMI 25 to < 30	212 (3.3%)	723 (12.0%)	1,461 (36.1%)	992 (32.4%)	424 (16.1%)	
BMI ≥ 30	395 (5.7%)	1,126 (20.3%)	1,673 (35.8%)	1,010 (26.9%)	379 (11.3%)	
SBP (mmHg)	132.2 ± 1.1	130.2 ± 0.6	127.7 ± 0.4	126.5 ± 0.5	125.8 ± 0.7	< 0.001
DBP (mmHg)	70.9 ± 0.7	71.9 ± 0.4	72.0 ± 0.3	70.5 ± 0.3	70.5 ± 0.4	< 0.001
Smoking status						< 0.001
Never smoker	323 (3.9%)	1,111 (14.6%)	1971 (33.6%)	1,397 (30.9%)	619 (16.9%)	
Former smoker	212 (3.3%)	655 (12.9%)	1,356 (34.1%)	990 (32.9%)	432 (16.8%)	
Current smoker	251 (9.2%)	564 (21.6%)	650 (32.8%)	398 (24.6%)	181 (11.8%)	
Diabetes						< 0.001
No	562 (4.2%)	1714 (14.3%)	3,090 (33.6%)	2,197 (31.0%)	984 (16.9%)	
Yes	226 (6.4%)	619 (19.5%)	888 (33.6%)	589 (28.5%)	250 (12.0%)	
Hypertension						< 0.01
No	311 (3.7%)	1,096 (15.1%)	1915 (34.2%)	1,274 (31.4%)	531 (15.7%)	
Yes	477 (5.5%)	1,237 (15.4%)	2063 (33.1%)	1,512 (29.6%)	703 (16.4%)	
CVD						< 0.001
No	641 (4.1%)	2022 (14.9%)	3,474 (33.9%)	2,432 (30.5%)	1,090 (16.5%)	
Yes	147 (8.1%)	311 (18.0%)	504 (31.4%)	354 (30.3%)	144 (12.2%)	
Stroke						< 0.05
No	719 (4.4%)	2,188 (15.1%)	3,780 (33.8%)	2,633 (30.6%)	1,158 (16.0%)	
Yes	69 (8.2%)	145 (17.3%)	198 (30.3%)	153 (28.3%)	76 (15.8%)	
Survey cycles						< 0.001
2007–2008	190 (6.0%)	511 (18.1%)	813 (37.4%)	495 (30.5%)	116 (8.0%)	
2009–2010	184 (5.8%)	508 (15.3%)	900 (36.5%)	583 (29.9%)	217 (12.5%)	
2011–2012	136 (3.6%)	455 (16.3%)	678 (30.4%)	540 (30.2%)	273 (19.4%)	
2013–2014	152 (4.7%)	417 (14.6%)	755 (32.1%)	612 (31.1%)	314 (17.6%)	
2015–2016	126 (3.3%)	422 (12.8%)	812 (32.9%)	556 (30.8%)	314 (20.3%)	

# Non-single: married or living with a partner; single: widowed/divorced/separate/never married.

Continuous variables were presented as weighted mean ± standard error. Categorical variables were presented as count (weighted percentages), with row counts summing to 100%.

PIR, ratio of family income to poverty; BMI, body mass index; SBP, systolic blood pressure; DBP, diastolic blood pressure; CVD, cardiovascular disease.

By multiple linear regression analysis, our results showed that older age, female, white people, non-smokers, higher educational attainment, married or living with partners, and higher income correlated to higher serum vitamin D levels, while individuals with CVD and higher BMI were associated with lower vitamin D levels (Table 2).

**Table 2:** Factors associated with serum vitamin D level by multiple linear regression.

Parameter	Estimate (Beta)	95% CI lower bound	95% CI upper bound	*p*-value
Intercept	62.60	53.47	71.74	< 0.001
Age	0.33	0.22	0.45	< 0.001
Female (vs. male)	7.84	5.98	9.70	< 0.001
Race/ethnicity				
White people	Reference			
Black people	−17.05	−19.55	−14.55	< 0.001
Hispanic	−11.37	−13.64	−9.09	< 0.001
Other	−9.23	−12.84	−5.61	< 0.001
BMI	−0.84	−0.99	−0.70	< 0.001
Month examination performed				
Nov 1 through Apr 30	Reference			< 0.05
May 1 through Oct 31	3.10	0.52	5.69	
Diabetes	0.33	−1.97	2.63	> 0.05
Hypertension	2.69	0.90	4.47	< 0.01
CVD	−3.39	−5.48	−1.31	< 0.01
Stroke	1.57	−2.72	5.87	> 0.05
Smoking status				
Current	Reference			
Never	4.82	2.65	6.99	< 0.001
Former	6.31	3.67	8.94	< 0.001
Educational attainment				
Lower (≤ high school)	Reference			
Higher (≥ some college)	2.07	0.13	4.01	< 0.05
Marital status^#^				
Single	Reference			
Non-single	1.77	0.15	3.39	< 0.05
PIR	1.88	1.22	2.55	< 0.001

# Non-single: married or living with a partner; single: widowed/divorced/ separate /never married.

PIR, ratio of family income to poverty; CVD, cardiovascular disease; BMI, body mass index.

**Figure 3:**
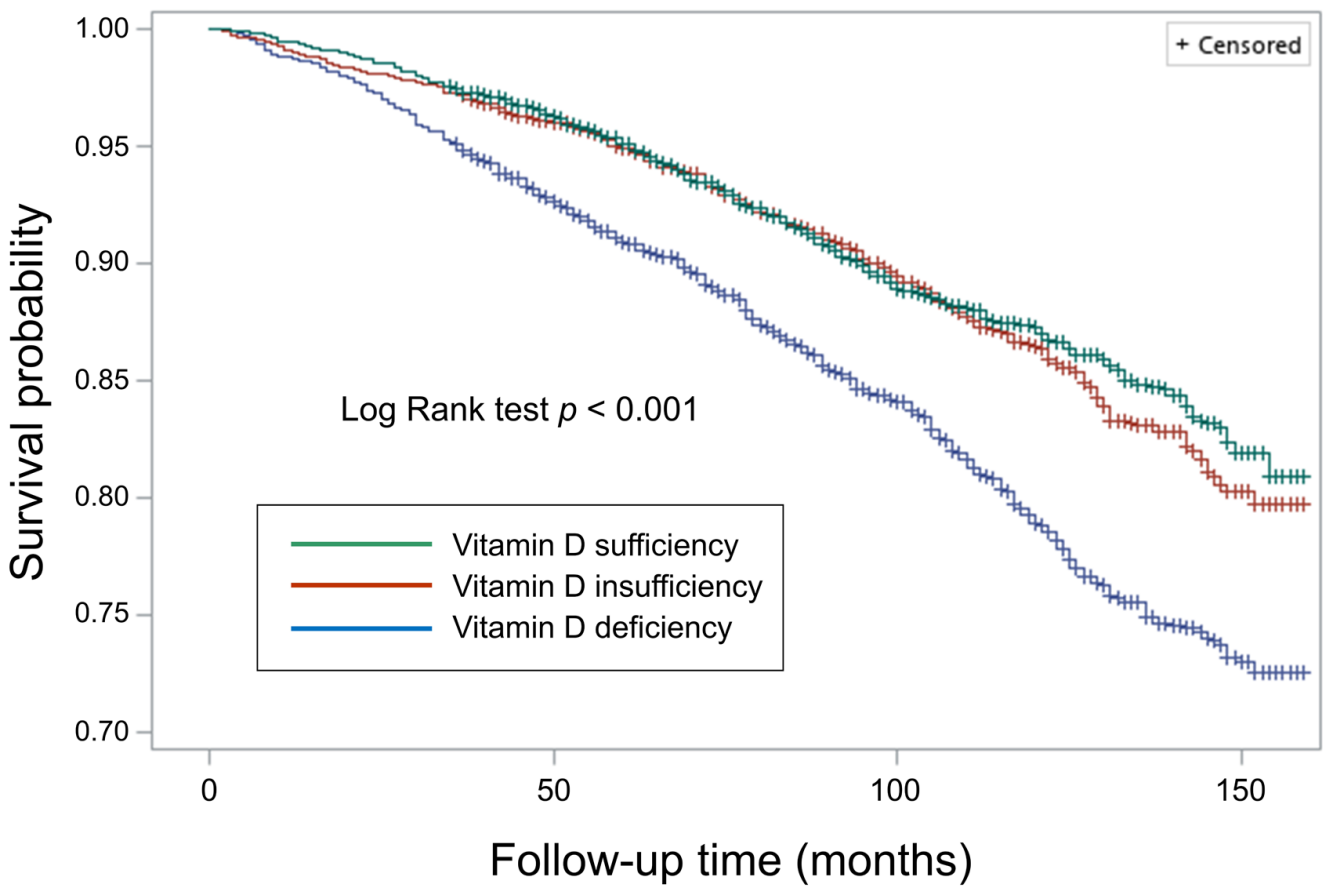
Survival curves for all-cause mortality by weighted Kaplan–Meier method with Log Rank test.

**Figure 4:**
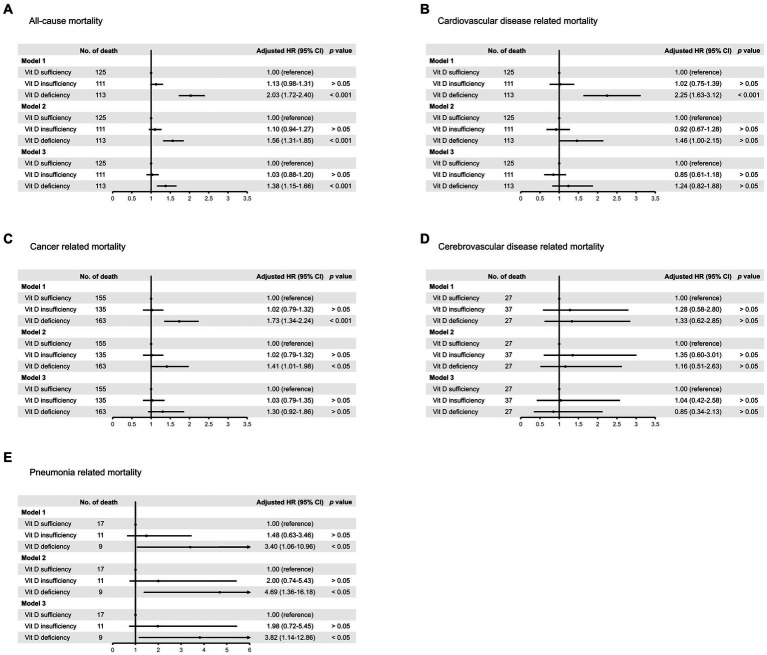
Risk of death from **(A)** all causes, **(B)** cardiovascular disease, **(C)** cancer, **(D)** cerebrovascular disease, and **(E)** pneumonia, among individuals with vitamin D deficiency or insufficiency compared with those of vitamin D sufficiency. Regression model 1 was adjusted for age, sex, and survey months. Model 2 was adjusted for variables in model 1 plus body mass index, diabetes, hypertension, cardiovascular disease, previous stroke, and smoking status. Model 3 was adjusted for variables in model 2 plus marital status, educational attainment, and family income to poverty ratio.

During a median follow-up of 97.0 months (interquartile range: 68.0–125.0 months), a total of 1,585 participants died (15.9 per 10,000 person-months). We observed that individuals with vitamin D deficiency (crude HR: 1.67, 95% CI 1.39–2.02, *p* < 0.001), but not those with vitamin D insufficiency (crude HR: 1.06, 95% CI 0.91–1.22, *p* > 0.05), were at higher risk of death from all causes compared with those who were sufficient in vitamin D level (Figure 3). The association remained similar after adjusting for age, sex, survey months, BMI, diabetes, hypertension, CVD, previous stroke, smoking status, and socioeconomic factors including marital status, educational attainment, and family income to poverty ratio (model 1 through model 3 in Figure 4A).

Regarding cause-specific mortality risk, we observed that vitamin D deficiency correlated to increased risk of death from CVD (HR: 2.25, 95% CI 1.63–3.12), cancer (HR: 1.73, 95% CI 1.34–2.24), and pneumonia (HR: 3.40, 95% CI 1.06–10.96), but not CVA after adjusting for age, sex, and survey months (model 1 in Figures 4B–E). However, after further adjustment for potential confounders, the risk of death from CVD or cancer associated with vitamin D deficiency was attenuated, but the association remained significant for pneumonia-related mortality (model 2 and model 3 in Figures 4B–E).

When compared with those of vitamin D sufficiency, we showed that individuals with vitamin D deficiency tended to be younger, male, non-white people, overweight or obese, smokers, single, of lower educational attainment, of low or middle income, and having CVD by multinomial logistic regression analysis (Table 3). Furthermore, younger participants, male, non-white people, those who were overweight or obese, or of low or middle income were more likely to be vitamin D insufficient (Table 3).

**Table 3:** Predictors of vitamin D deficiency and vitamin D insufficiency compared with vitamin D sufficiency.

	Vitamin D deficiency	Vitamin D insufficiency
	OR (95% CI)	OR (95% CI)
Age group		
50–59	1.94 (1.55–2.42)^***^	1.58 (1.36–1.83)^***^
60–69	1.33 (1.04–1.69)^*^	1.13 (0.96–1.34)
70–79	1	1
Sex		
Male	1.30 (1.10–1.53)^**^	1.70 (1.47–1.97)^***^
Female	1	1
Race/ethnicity		
White people	1	1
Black people	5.31 (4.22–6.67)^***^	1.76 (1.47–2.10)^***^
Hispanic	3.23 (2.55–4.08)^***^	2.39 (2.04–2.81)^***^
Other	2.72 (1.81–4.10)^***^	1.71 (1.22–2.41)^**^
Survey months		
November–April	1.65 (1.35–2.03)^***^	0.94 (0.81–1.09)
May–October	1	1
BMI status		
Normal	1	1
Overweight	1.46 (1.20–1.77) ^***^	1.63 (1.37–1.92) ^***^
Obese	3.27 (2.61–4.10) ^***^	2.18 (1.77–2.69) ^***^
Smoking status		
Never smoker	0.54 (0.45–0.65) ^***^	0.91 (0.75–1.09)
Former smoker	0.48 (0.39–0.58) ^***^	0.84 (0.66–1.07)
Current smoker	1	1
Marital status		
Married/with a partner	1	1
Unmarried/widowed/divorce	1.38 (1.16–1.65) ^***^	1.05 (0.87–1.26)
Educational attainment		
≤ high school	1.24 (1.03–1.49) ^*^	1.04 (0.90–1.20)
≥ some college	1	1
Family income to poverty ratio		
low income	1.79 (1.41–2.25) ^***^	1.59 (1.29–1.95) ^***^
middle income	1.44 (1.16–1.79) ^**^	1.32 (1.10–1.59) ^**^
high income	1	1
Diabetes	1.07 (0.86–1.33)	1.00 (0.82–1.22)
Hypertension	0.78 (0.67–0.91)^**^	0.88 (0.76–1.02)
Cardiovascular disease	1.38 (1.08–1.75)^**^	0.96 (0.79–1.17)
Stroke	0.97 (0.67–1.39)	0.87 (0.66–1.16)

^*^p < 0.05; ^**^p < 0.01; and ^***^p < 0.001.

## Discussion

Among non-institutional United States middle-aged and older adults, we observed that more than half of them were vitamin D deficient or insufficient, but the secular trend of vitamin D deficiency or insufficiency was declining. The serum vitamin D levels positively correlated to older age, female, white people, non-smoker, higher educational attainment, married or living with partners, and higher income and negatively correlated to BMI. Additionally, we showed that vitamin D deficient individuals were more likely to be younger, male, non-white people, overweight or obese, smokers, single, less educated, and had low or middle income. Furthermore, our results showed that individuals with vitamin D deficiency, but not those with vitamin D insufficiency, were associated with a higher risk of all-cause and pneumonia-related mortality.

The recommended thresholds to define vitamin D deficiency have been under debate (15). The Institute of Medicine defined serum level of < 30 nmol/L as vitamin D deficiency and of 30–50 nmol/L as vitamin D insufficiency (17), while the Endocrine Society defined deficiency and insufficiency by vitamin D level of < 50 nmol/L and 50–75 nmol/L, respectively (16). Determining the cutoff levels to define vitamin D deficiency and insufficiency is of clinical importance for assessing the health outcomes associated with these conditions (18). Our study used thresholds recommended by the Endocrine Society (16) and showed that a vitamin D level of < 50 nmol/L, but not a vitamin D level of 50–75 nmol/L, was associated with a higher risk of death compared with a vitamin D level of > 75 nmol/L. Our results may have clinical implications for determining recommended vitamin D thresholds.

Vitamin D deficiency is a common global public health issue (19, 20), as a growing body of literature has highlighted its association with several acute and chronic health conditions. However, the reported prevalence of vitamin D deficiency varied in different populations and regions worldwide (21, 22). In South Asia, the overall prevalence of vitamin D deficiency was 68%, with substantial heterogeneity among populations (23). In Indian adults older than 50 years, community-based studies reported a prevalence of vitamin D deficiency (< 50 nmol/L) higher than 50% (24). In addition, among European adults aged older than 50 years, the prevalence of vitamin D levels of < 30 nmol/L and < 50 nmol/L were 10–20% and 40–50%, respectively, based on population-based studies (9–11, 25, 26). Contrary to these reports, our data showed a lower prevalence of vitamin D deficiency in United States adults aged 50 years or above, nearly 20%. Several factors, such as latitude, sun exposure, and vitamin D photosynthetic response, have been linked to vitamin D status (20). In addition, the use of vitamin D supplements and the availability and affordability of vitamin D-rich foods may also be important factors contributing to the results (11, 20). Our study found that non-white participants and those of lower socioeconomic status were more likely to have vitamin D deficiency or insufficiency, suggesting that social determinants of health may play roles in serum vitamin D levels (9, 10, 27). Regardless, while the risk of vitamin D deficiency may be related to multiple issues, strategies are needed to narrow the gap between populations to reduce health disparities (20).

Numerous observational studies have shown that vitamin D levels inversely correlated to the risk of several chronic health conditions such as cancer, cardiovascular disease, diabetes, cognitive decline, and depression (2, 28), while large-scale randomized trials have generally demonstrated the null effects of vitamin D supplementation on these health outcomes (28). Our study showed that vitamin D deficiency was associated with an increased risk of all-cause mortality. However, the associations between vitamin D and the risk of death from CVD or cancer were attenuated after adjusting for socioeconomic factors, suggesting that there may not be a causal relationship between vitamin D and these health outcomes. It has been suggested that a variety of medical and non-medical factors may be involved. For example, people with higher vitamin D levels may have healthier lifestyles (11) and better access to healthcare (27), reducing their risk for adverse health consequences. Taken together, low vitamin status may be an indicator of, rather than a contributing factor to, poor health.

Vitamin D has been reported to modulate immune responses by inducing monocyte differentiation, stimulating phagocytosis-dependent and antibody-dependent macrophages, and modulating cytokine and antibody-producing lymphocytes (29–32). In addition, vitamin D may facilitate the production of cathelicidin, an antimicrobial peptide whose expression increases on immune cells in response to infection (32–34). Several studies have identified low vitamin D status as a risk factor for respiratory tract infections (35–38) and worse clinical outcomes (39–42). However, there were conflicting findings on the beneficial effects of vitamin D supplementation in patients with respiratory tract infections or pneumonia (42–45) This sparked a debate about whether the association between vitamin D and pneumonia might be due to reverse causation, whereby lower vitamin D levels in acute inflammatory states are directly proportional to disease severity (28, 46–49). Nevertheless, a meta-analysis of observational studies demonstrated a non-linear inverse relationship between vitamin D concentration and the risk of future respiratory tract infection in healthy adults or adolescents, with the greatest increased risk for vitamin D concentrations of < 37.5 nmol/L (50). Our findings added to the evidence supporting the association between vitamin D status and respiratory tract infection, showing that vitamin D deficiency (< 50 nmol/L), but not vitamin D insufficiency (50.1–70 nmol/L), correlated to the future risk of dying from pneumonia.

Using a nationally representative sample of the noninstitutionalized United States civilian population, our study demonstrated the prevalence and risk factors of vitamin D deficiency and its association with adverse health outcomes. However, there were several study limitations that should be considered. First, in addition to ultraviolet B availability and dietary vitamin D supply, multiple factors can contribute to vitamin D statuses, such as working environment, outdoor physical activity, personal skin pigmentation, sun-screen usage, and other sun protective behaviors (20, 51, 52). Additionally, various vitamin D supplements with different proportions of intestinal absorption and bioavailability may also lead to differences in vitamin D levels (53). While these variables were not recorded in our study, we were unable to assess the associations between these factors and vitamin D status. However, this did not affect the assessment of the association between serum vitamin D status and mortality risk. Second, vitamin D levels may change over time and seasonal variations may occur (52). Classifying participants by a single baseline recording could lead to misclassification bias. Third, while 25-hydroxyvitamin D levels may not reflect the biologically active or free fraction of vitamin D, using this parameter to define vitamin D status may have led to information bias. Fourth, because comorbidities and socioeconomic variables were ascertained by participants’ self-report, information bias possibly impacts the validity of the study results. Finally, although we have adjusted for several potential confounders, residual confounding factors may exist. For example, individuals with low vitamin D levels may have unhealthy lifestyles that predispose them to adverse health outcomes. Given the observational nature of our study, a causal relationship between vitamin D status and risk of death could not be established.

In summary, among middle-aged and older adults in the US, one-fifth of them were vitamin D deficient, but the secular trend was improving. While vitamin D deficiency, but not vitamin D insufficiency, is associated with an increased risk of all-cause and pneumonia-related mortality, future research is needed to explore the potential causal relationship and underlying mechanisms.

## Data availability statement

Publicly available datasets were analyzed in this study. This data can be found at: https://wwwn.cdc.gov/nchs/nhanes/Default.aspx.

## Ethics statement

Ethical review and approval was not required for the study on human participants in accordance with the local legislation and institutional requirements. The patients/participants provided their written informed consent to participate in this study.

## Author contributions

M-YJ and T-YW: conceptualization and funding acquisition. M-YJ: methodology, software, formal analysis, investigation, resources, data curation, writing—review and editing, visualization, supervision, and project administration. M-YJ, H-WW, and T-YW: validation. T-YW and H-WW: writing—original draft preparation. All authors contributed to the article and approved the submitted version.

## Funding

This study was funded by the Chi Mei Medical Center in the form of a grant to Hung-Wei Wang [CMFHR11077]. The APC was funded by Chi Mei Medical Center.

## Conflict of interest

The authors declare that the research was conducted in the absence of any commercial or financial relationships that could be construed as a potential conflict of interest.

## Publisher’s note

All claims expressed in this article are solely those of the authors and do not necessarily represent those of their affiliated organizations, or those of the publisher, the editors and the reviewers. Any product that may be evaluated in this article, or claim that may be made by its manufacturer, is not guaranteed or endorsed by the publisher.

## Footnotes

^1^ https://wwwn.cdc.gov/nchs/nhanes/tutorials/module2.aspx
